# Monthly Summary

**Published:** 1895-10

**Authors:** 


### Monthly Summary.

PORCELAIN-FACED BICUSPID CROWNS.—Regarding the making of these crowns, Dr. A. W. NcCandless says: "The root having been properly repaired, a band is fitted thereto exactly as though an ordinary telescope or shell gold crown were to be made.

The buccal side of this ferrule is cut out with curved shears at about where the porcelain facing is to be placed. Secure a perfect articulation in the usual manner, and then tack the cusp to the ferrule at a point the farthest from the porcelain; now, with the curved shears, cut away the gold of the cusp down to the point of the cusp, approximating the shape of the porcelain face at the end. Grind the facing, and trim the opening in the gold that is to receive it, until the fit is fairly accurate, but not absolutely so, however, as this is entirely unnecessary, and requires too much time.

When the facing has been ground to the proper shape, remember to always make it a trifle smaller than the space, to allow for the thickness of the gold backing, bevel the edges of the porcelain all the way around, then when the gold backing is thoroughly fitted, which is only accomplished by thorough painstaking, annealing the gold often, and gently tapping to place with a small riviting hammer, place the tooth, backed with pure gold of thirty gauge, in position. Catch the gold backing to the ferrule and cast with sticky wax, slip the porcelain off, invest the gold in long fiber asbestos, moistened with water, leaving only enough gold exposed so that with a small particle of solder, the backing may be united to the ferrule, remove the investment, fill the joints where the solder is to flow, with wax, fill the entire interior of the crown with a paste of plumbago, or stove polish, so that the solder will *not* flow where you would not have it go, then complete the soldering of the joints and flow up sufficient solder to give the proper contour to the finished crown. At this point a lower grade of solder is used for filling the cusps. The porcelain facing is slipped to place, the gold filed or ground to the proper shape and finished, and if the work has been properly done up to this point, a perfect joint between backing and tooth is the result. Remove the facing, then with a thin film of cement between the gold and the porcelain, force the facing to place.

After the cement has set bend the pins of the tooth carefully outward from each other, and the facing is held with sufficient strength. In making these crowns 22 carat gold, 28 guage and 22 carat solder is used, with 14 carat solder in the cusps.

After the crown has been finished and polished send it to a gold plater, who for ten cents will plate it with pure gold, which gives it a very beautiful appearance.

The plating is not designated to cover any pits or other defects caused by faulty manipulation, as it brings them out and magnifies, them but a crown thus treated will not tarnish in the mouth and the plating is quite durable where no friction comes, and where friction is the tarnish will not come.

There are a good many things to commend this form of crown—natural appearance, strength and utility. No risk is incurred in soldering, the color preserved, permitting more artistic effect in matching the natural teeth and the absence of that blue line so common at the cervical margin of soldered teeth."—*Detnal Review.*

---

THE IMPORTANCE OF PROSTHETIC DENTISTRY.—In his essay on "Dental Furnaces" before the Odontological Society of Pennyslvania, Dr. M. W. Sharp makes a strong protest against the belittling of prosthetic dentistry, and the giving the impression that operative dentistry requires greater skill and is necessarily of greater importance than the mechanical. This tendency will be readily seen in the contrast noticeable in many offices between the operating room and the laboratory—the one furnished with every appliance known to modern dentistry, the other bare of everything more modern than lathe, plaster can and vulcanizer. The unpleasant, meagre furnishing of the laboratory has much to do with this feeling. If we would elevate mechanical dentistry we must attempt more difficult operations. There is here a wide field for investigation and improvement, and to one sufficiently of a mechanical turn of mind to becomb a successful dentist there should be facination when once engaged. There is really nothing unpleasant about the mechanical work and fees are relatively much higher—*Dominion Dental Journal.*

---

FITTING CROWNS.—Dr. Bryan, in the *International*, gives the following method of preparing an opaque black wax for articulating crowns. One part by bulk of lamp black and five parts white or yellow wax are melted together and rolled into sticks. The crown and root being ground to fit approximately, a small piece of this wax warmed is placed around the pin and the crown placed in position. The points requiring grinding will be accurately indicated. The amount to be removed being estimated by "sounding" the wax with a fine point.

---

BEADED ENCLOSURES IN VULCANITE DENTURES.—W. Storer How, D. D. S., Philadelphia, advocates the making of a "beaded enclosure" on vulcanite dentures to take the place of the vacum chamber so generally used. The beaded enclosure is made by scraping a suitable smooth half round groove in the plaster model of any size or shape deemed advisable. A similar groove across the palatal portion of the model and along the buccal and labial lines of muscular attachment will produce a supplemental chamber-like function of the entire surface of the denture.—*Dental Cosmos.*

CHRONIC ALVEOLAR ABSCESS WITH COMPLICATIONS.—Dr. Truman W. Brophy, in a paper under this heading in the *Dental Review*, makes an instructive tabulation of the complications to be met with in practice. Abscesses failing to respond to ordinary careful treatment are pretty certain to have some complication. It may be in the form of an apex denuded of its pericementum by suppuration. In many cases the fistula may be at a most unexpected point, as into the nasal passage or antrum in case of an upper tooth, or as low down as the clavicle or the nipple in case of a lower. Where the pus enters and fills up the antrum of high more the opening into the nasal passage may close and the floor of the orbit be pressed up, and great pain be caused by pressure on the infraorbital nerve, or pus may dribble from the eye. This condition may deceive even a skilled ophthalmologist. Pus does not always escape at point of lowest resistance, as for example pus formed at roots of incisor teeth may burrow along to hard palate, loosening the periosteum from bone, which condition is very dangerous as it may result in necrosis or caries of bone. Another complication frequently met is where the pulp dies in one or more roots of a tooth, and remains alive in another. The live portion of the pulp responding to heat applied as a test will mislead the operator unless he is on the lookout for such a condition. In concluding his paper Dr. Brophy makes a plea for very careful diagnosis, as very serious results may come from alveolar abscess.—*Dominion Dental Journal.*

---

PULP CAPPING.—Use gum dammar, cover this with oxyphosphate of zinc. When hard fill the tooth with what you like.—*Dental Digest.*

---

FOR SETTING CROWNS.—W. H. Rollins recommends one part gutta percha and three parts vermillion, mixed with heat and careful working, for setting crowns, the combination being strongly resistant to the fluids of the mouth.—*Southern Dentas Journal.*

ARGONIN.—A. Liebrecht, of Breslan (*Ther. Monats*, June, 1895), has given the name of *argonin* to a combination of *nitrate of silver* mixed with a *sodium* composed of *casein* and precipitated by *alcohol*. Mayer finds that it is easily soluble in hot water, and with difficulty in cold water, but a 10-per-cent. solution can be prepared. It is readily soluble in *albumen*, and a 10 per-cent. solution in *serum* can be made. It is of considerable strength as an antiseptic, though the watery solutions are less powerful than *silver nitrate* or the albuminous solutions. It does not irritate the mucous membrane.

---

OF ALL pauperism, the most degraded and degrading, because utterly shameless aand thriftless, is that aristocracy which idly luxuriates in money obtained through speculation, extortion or inheritance.—*Items of Interest*.

---

A VENT IN YOUR PORT POLISHER.—By Earl D. Eddy, San Francisco.—I have just been drilling a hole in the socket of a plain port polisher. As I was in the act of making this improvement, the thought occurred to me that the makers ought to do this when the instrument is being constructed. I have never seen this improvement in any port polisher but my own, and thinking that you might use the idea for the benefit of the profession, I concluded to call your attention to the value of a vent for the escape of air in the base of the socket. This vent greatly facilitates the setting of the points, as there is no compressed air present to displace them when they are pressed in the socket.—*Items*.

---

OBTUNDING SENSITIVE DENTINE.—Dry the cavity thoroughly, using warm air and alcohol alternately. Then apply a 10 per cent. "chloroform solution of cocaine," which will be absorbed by the dentine, anæsthetizing it perfectly. Chloroform applied on cotton hastens the absorption of the cocaine.—*Items of Interest*.

ALCOHOLISM IN FRANCE.—A communication by Laborde served as the starting-point of a discussion on the prophylaxis of alcoholism. Rochard painted a vivid picture of the progress of the vice in France and its disastrous results, and proposed the re-establishment of the law of 1865. Lagneau and Lancereaux dwelt upon the relation of alcoholism to tuberculosis, and from the discussion it was evident that the members regarded it as a fact beyond doubt that the increase of the vice in question was in direct relation with the increase of consumption, particularly in men. Of 10,681 deaths from the disease registered in Paris in 1893, 6,553 were men and 4,128 women. As a prophylactic measure increased taxation of wine-shops was generally advocated, with restriction of the number of drinking-places, which in Paris alone is 34,500.

---

CYST OF THE ANTRUM OR HIGHMORE.—Otto Umgrem had under his care a baker, of thirty-five years, tall and thin, who for a long time had suffered from pain and swelling of the right cheek. Sensibility to pressure, however, was not increased. The tumor was hard and firm. Extraction of the first molar gave exit to a large quantity of sanguinolent pus having a bad odor, but not fetid. On sounding through the extraction wound, Umgren found a cavity the size of a small hen's egg, extending in various directions. There was no issue in the nasal cavity for the pus, which had accumulated in the antrum of Highmore.—*Journal Odontologique.*

---

MR. LAWSON TAIT is probably the most aggressive opponent of the germ theory of inflammation. He says that during his professional life he has learned and unlearned some some four or five theories of inflammation, and predicts that the present prevalent theory—a phase of lunacy; coccophobia —will soon go the way of the other theories—*Medical Age.*

